# Bio‐based and biodegradable plastics: An annotated selection of World Wide Web sites relevant to the topics in microbial biotechnology

**DOI:** 10.1111/1751-7915.13502

**Published:** 2019-10-20

**Authors:** Lawrence P. Wackett

**Affiliations:** ^1^ Department of Biochemistry, Molecular Biology and Biophysics, BioTechnology Institute University of Minnesota St. Paul MN 55108 USA

## Bio‐based and biodegradable plastics

https://waste-management-world.com/a/guest-blog-the-difference-between-biobased-biodegradable-plastics

The terms bio‐based plastics and biodegradable plastics are sometimes used interchangeably, but they are not the same. Bio‐based plastics derive from non‐petroleum biological resources. Biodegradable plastics degrade via exposure to naturally occurring microbes and may be bio‐based or made from petroleum.

## Bio‐based plastics

https://www.plasticseurope.org/en/about-plastics/what-are-plastics/large-family/bio-based-plastics

This review article discussed starch blends, poly‐lactic acid, bio‐polyethylene terephthalate, bio‐polyethylene and polymers from bio‐based succinic acid.

## Bioplastic

https://en.wikipedia.org/wiki/Bioplastic

This Wikipedia entry provides a fairly comprehensive overview of bio‐based plastics.

## Are bioplastics better for the environment

https://ensia.com/features/bioplastics-bio-based-biodegradable-environment/

This article provides a good overview of the source and production of bioplastics, and it also considers marketability of these materials.

## Bio‐based and compostable plastics

https://www.organixsolutions.com/compostable-bioplastics

This post describes both bio‐based and compostable (biodegradable) polymers. For example, the carbon in bio‐based plastics such as poly‐lactic acid is typically derived from corn starch. There are examples of petroleum based polymers that are biodegradable.

## Why is there a delay to use bio‐based plastics

https://www.plasticstoday.com/sustainability/study-asks-why-switch-biobased-plastics-taking-so-long/73993200161277

One highlight of this article is reference to a new market study on bio‐based polymers. Fundamentally, bio‐based polymers have not seen very widespread use due to cost and lack of manufacturing scale.

## Types of biodegradable plastic

https://greenliving.lovetoknow.com/Type_of_Biodegradable_Plastic

This popular press type article gives an excellent description of a range of biodegradable polymers.

## Designing bio‐based recyclable polymers

https://www.cell.com/trends/biotechnology/fulltext/S0167-7799(19)30089-7

This review article describes multiple bio‐based polymers and the role of microbial cells and enzymes in the production and recycling of polymers.

## Bioplastics: Markets and developments

https://www.labiotech.eu/features/bioplastics-2019-feature/

This article focuses on the total market for plastics, and the increment of bioplastics, both currently and in projected years.

## Bio‐based plastics with insect‐repellent functionality

https://onlinelibrary.wiley.com/doi/abs/10.1002/pen.25083

This paper describes a method for extrusion or spray coating of natural and synthetic insect repellents into poly‐lactic acid fibres.

## Bio‐based polymers: Department of Energy

https://www.energy.gov/eere/amo/plastics-or-fibers-bio-based-polymers

This page focuses on the energy and carbon emissions savings that can be attributed to the use of bio‐based polymers such as polylactic acid.

## A new industrial revolution for plastics

https://www.usda.gov/media/blog/2018/09/19/new-industrial-revolution-plastics

This optimistic article discussed the potential for increases in bio‐based plastics. It posits that the global bioplastic packaging market is anticipated to grow at a rate of 18% between 2017‐2025.

## Advances in biodegradable polymers: Special issue

https://www.mdpi.com/journal/molecules/special_issues/Biodegradable_Polymers

This link is to a special issue of the journal *Molecules*, focused entirely on different aspects of ‘Advances in Biodegradable Polymers’.

## Durability of biodegradable polymers

https://www.frontiersin.org/articles/10.3389/fmats.2019.00151/full

This article specifically focuses on the use of biodegradable polymers for applications in conservation of cultural heritage materials.

## Biodegradable polymers and their biomedical applications

https://www.degruyter.com/view/j/epoly.2019.19.issue-1/epoly-2019-0041/epoly-2019-0041.xml

Biodegradable polymers have important applications for controlled delivery of drugs, or in sutures where only limited material lifetime is desirable.

## Cyclopropenium‐based biodegradable polymers

https://pubs.acs.org/doi/10.1021/acs.macromol.9b00430

Cyclopropenium‐based biodegradable polymers are useful cationic polymers that traditionally have not been viewed as biodegradable. The current report describes the synthesis of biodegradable members of this class.

## Biodegradable polymers list of high impact articles

http://www.imedpub.com/scholarly/biodegradable-polymers-journals-articles-ppts-list.php

This webpage provides a compendium of recent journal articles related to biodegradable polymers.

## Center for Sustainable Polymers

https://csp.umn.edu

The Center for Sustainable Polymers is funded by the United States National Science Foundation and is committed to research, education and public outreach related to the sustainable production and use of polymeric materials.

